# Supplementary material for: A dressed singlet-triplet qubit in germanium
Source: Nat Commun. 2026 Jan 20;17:699. doi: 10.1038/s41467-025-65569-3 (PMC12820228; doi:10.1038/s41467-025-65569-3)
Supplement: Supplementary file 1 — Supplementary Information [file 41467_2025_65569_MOESM1_ESM.pdf]

Supplementary information for:
A dressed singlet-triplet qubit in germanium

A. INITIALIZATION AND READOUT OF TWO SPINS

Spin-to-charge conversion is employed to distinguish the spin states of the double quantum dot by mapping them to specific I_{sensor} values. The asymmetry of the loading rates of QD1 and QD2 is leveraged for this process, with QD1 loading much faster due to its proximity to the hole reservoir (O1), as can be seen in Fig. 1a of the main text. This is the well known enhanced latching readout. In Fig. S1a, we illustrate the pulse sequence that allows us to map out the readout window in the charge stability diagram (CSD). The spins are initialized in the $|\downarrow\downarrow\rangle$ state (discussed in detail in the third paragraph) at point 1. We then apply either an identity gate (left panel), thus remaining in the $|\downarrow\downarrow\rangle$ state, or perform a spin flip on Q1 (right panel), thus transitioning to the $|\widetilde{\uparrow\downarrow}\rangle$ state, before finally pulsing adiabatically to point 2. There, the $|\widetilde{\uparrow\downarrow}\rangle$ state will be blockaded, resulting in a (1,1) charge state, while the $|\downarrow\downarrow\rangle$ state will allow interdot tunneling resulting in a (2,0) charge state (discussed in detail in the third paragraph). Subsequently, by pulsing across the fast (1,0)$\leftrightarrow$(2,0) transition, a second charge is loaded into QD1 while the hole under QD2 remains trapped, as it cannot unload through the reservoir or the interdot. Therefore, the blockaded state maps to the (2,1) configuration, allowing for long integration times ($50\,\mu$s) and an enhanced signal. The readout point (R) is chosen in the center of the readout window to increase robustness against drifts of the CSD.

In systems using Pauli spin blockade, initialization and readout are strongly dependent on the ramp rate between the (2,0) and (1,1) charge regions. This is mostly related to the $\mathrm{S}(2,0)$-$\mathrm{T}_-(1,1)$ anticrossing, as shown in Fig. S1b. This anticrossing is measured in Fig. S1c, where starting from point T (Fig. 1b of the main text), we apply a square pulse of varying amplitude ε and wait time (t_{wait}), before pulsing to R to read out the spin state. The frequency of the resulting spin oscillations maps out the $\mathrm{S}(2,0)$-$\mathrm{T}_-(1,1)$ energy difference with a minimum observed at the $\mathrm{S}(2,0)$-$\mathrm{T}_-(1,1)$ anticrossing.

To initialize the system, we introduce a ramp to the detuning pulse to ensure adiabatic passage through all transitions (super slow adiabatic passage). Fig. S1d shows that for ramp times longer than 500 ns, the initial oscillation pattern disappears, giving rise to a t_{wait}-independent I_{sensor} signal corresponding to a blockaded state. This indicates proper initialization into the $|\downarrow\downarrow\rangle$ state achieved by the slow ramp. Finally, we note that the rapid return pulse to the interdot results in the diabatic crossing of the $\mathrm{S}(2,0)$-$\mathrm{T}_-(1,1)$ anticrossing for all states. This maps the $|\downarrow\downarrow\rangle$ state to the (2,1) charge configuration instead.

After setting the initialization time at $t_{\text{ramp-in}} = 1\,\mu$s to ensure super slow adiabatic passage, we study the readout ramp time ($t_{\text{ramp-out}}$). We pulse to the center of the (1,1) configuration and apply a chirped pulse to explore the excited states of the two-spin system as a function of ramp-out time ($t_{\text{ramp-out}}$). As shown in Fig. S1e, when $t_{\text{ramp-out}} = 0$, the only non-blockaded state is $|\uparrow\downarrow\rangle$, which crosses the $\mathrm{S}(2,0)$-$\mathrm{T}_-(1,1)$ anticrossing diabatically. In contrast, for $t_{\text{ramp-out}} > 200\,$ns, the non-blockaded state is $|\downarrow\downarrow\rangle$, as it adiabatically crosses the same anticrossing. Consequently, with symmetric ramp times ($t_{\text{ramp-out}} = t_{\text{ramp-in}} = 1\,\mu$s), the two antipolarized states are mapped to the (2,1) charge configuration, distinguishing them both from the $|\downarrow\downarrow\rangle$ state. Unless otherwise specified, a symmetric $1\,\mu$s ramp was used to ensure super slow adiabatic passage (super SAP) for both initialization and readout.

We employ the same chirped pulse applied at the center of the (1,1) region to investigate the transition frequencies of the two-spin system. By varying the in-plane external magnetic field and monitoring the excitation frequencies of both spins, we estimate the g-factor for each qubit. As shown in Fig. S1f, the g-factors are determined to be 0.41 for Q1 and 0.44 for Q2, consistent with previously reported values for in-plane B fields in Ge/SiGe heterostructures.

B. SINGLE SPIN GATES AND SEQUENCES

To measure in the basis of the ST qubit, we apply an additional $\mathrm{X}_\pi^{\mathrm{Q1}}$ pulse, flipping the spin in QD1, as shown in Fig. S2a. This operation effectively distinguishes the $|\widetilde{\uparrow\downarrow}\rangle$ state from all other states.

The calibration of single-qubit gates (X_π, $\mathrm{X}_{\pi/2}$) for all qubits (Q1, Q2, ST, and dressed ST) follows a systematic procedure. First, the drive signal is applied on resonance at a frequency identified from the Rabi chevron pattern and its Fourier transform. Next, a two-dimensional scan is performed: the duration of the drive pulse is varied along one axis, while the number of consecutive pulses is varied along the other axis. The drive pulse induces a rotation of the state on the Bloch sphere, resulting in periodic oscillations with a characteristic speed calculated as $4/(\#\text{ of }\mathrm{X}_{\pi/2})$.

An oscillation speed of 0.5 corresponds to an X_π gate, while an oscillation speed of 0.25 corresponds to an $\mathrm{X}_{\pi/2}$ gate. This process is illustrated in Fig. S2b. The upper panel shows the measured signal after a specific number of

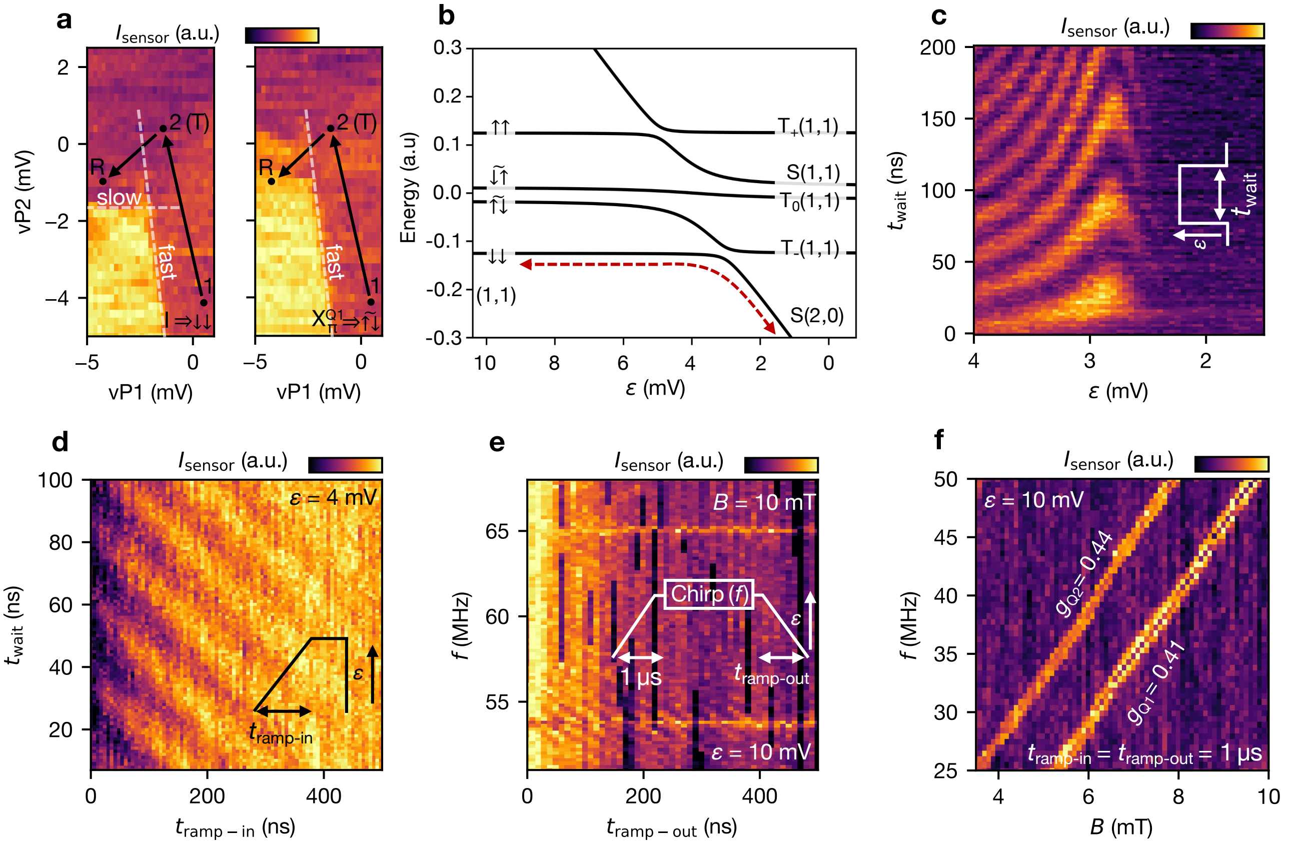

Figure S1. **Initialization and readout. a.** The $|{\downarrow\downarrow}\rangle$ state is initialized at point 1. Next we apply an identity I (do nothing for no time) (left panel) or an X_π^{Q1} pulse (right panel) to Q1, then cross to point 2 (point T in the main text), then sweep the readout point R. We observe a difference in I_{sensor} in a region above the (2,1) charge configuration, in the middle of which we place the R point. The ramp time between points 1↔2 was set at $t_{\text{ramp}} = 1\,\mu$s. Current is plotted in arbitrary units (a.u.). **b.** Energy diagram of the DQD spin system. The red arrow indicates the desired initialization (and readout) process, with a super slow adiabatic passage through the $\mathrm{S}(2,0)$-$\mathrm{T}_-(1,1)$ ensuring initialization (and readout) of the $|{\downarrow\downarrow}\rangle$ state. **c.** Spin oscillations originating from a square detuning pulse, mapping out the $\mathrm{S}(2,0)$-$\mathrm{T}_-(1,1)$ energy difference. **d.** By introducing a ramp at the beginning of the (2,0)-(1,1) detuning pulse, we observe that the oscillations disappear, indicating successful slow adiabatic passage through the $\mathrm{S}(2,0)$-$\mathrm{T}_-(1,1)$ anticrossing. **e.** By applying a chirped drive pulse, and adding a ramp in the return pulse to the (2,0), we confirm the selection rules of the spin-to-charge conversion process. By operating with a symmetric (in and out) ramp time of $1\,\mu$s, we ensure initialization and readout of the $|{\downarrow\downarrow}\rangle$ state. **f.** Monitoring the spin transition frequency (via a chirped pulse) while varying B allows us to extract the g-factor of each spin.

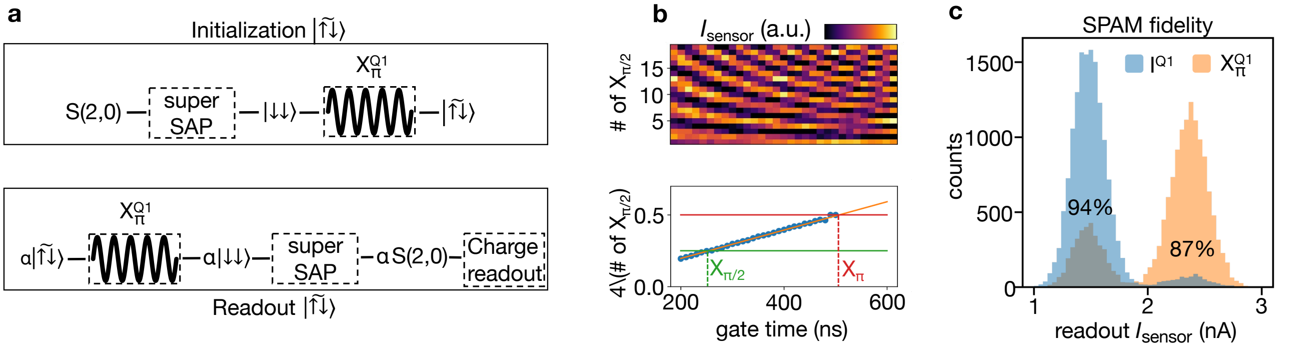

Figure S2. **Single spin initialization and readout fidelity. a.** Initialization and readout sequences of the $|\widetilde{\uparrow\downarrow}\rangle$ state. **b.** Calibration of the timing of single qubit gates. (Upper) Drive pulse duration versus the number of consecutive $\pi/2$ pulses applied. (Lower) Fourier transform revealing the oscillation speed, in units of number of $\pi/2$ pulses, from where the duration of a full and half rotation can be extracted. Current is plotted in arbitrary units (a.u.). **c.** Initialization and readout (SPAM) fidelity estimation after performing and I and X pulse on Q1.

gate repetitions as a function of the pulse duration. The lower panel displays the fitted oscillation speed for each pulse duration. A linear fit to the oscillation speed as a function of pulse duration enables the extraction of the X_π and $X_{\pi/2}$ durations, corresponding to oscillation speeds of 0.5 and 0.25, respectively. The fact that the duration of the $X_{\pi/2}$ gate is not exactly half that of the X_π gate may originate from various sources, including non-ideal pulse shaping (e.g., finite rise times) and drive-duration-dependent effects such as AC Stark shifts or local heating.

To evaluate the state preparation and measurement fidelity (SPAM), we perform a histogram analysis of 1000 shots for either an identity operation (I) or for an X_π gate on qubit 1. The resulting histogram, shown in Fig. S2c, reveals two well-separated Gaussian peaks corresponding to the $|{\downarrow\downarrow}\rangle$ and $|\widetilde{\uparrow\downarrow}\rangle$ states, demonstrating that the device could allow for operation in the single-shot readout regime. The fidelity after applying a (1 ns long) I gate is estimated to be 94 %. The fidelity after applying an X_π is estimated to be 87 %, with the lower fidelity for the X_π gate possibly attributed to relaxation during the longer gate time (500 ns), potential over-rotations and, more probably, decay inside the PSB window. A more detailed analysis of the magnitude of SPAM errors and the optimization of readout mechanisms is presented in Kelly *et al.* [28].

C. RESONANT EXCHANGE INTERACTION

In the middle of the (1,1) charge region, J can be approximated by $\frac{4t_c^2}{U}$, where U denotes the charging energy and t_c the quantum tunneling amplitude through the potential barrier separating QD1 and QD2. Therefore, the exponential dependence of t_c on the barrier potential will result in an exponential relation of J on vB12. By applying a sinusoidal modulation on the barrier gate $\mathrm{vB12}_{\mathrm{drive}} = \mathrm{vB12}_{\mathrm{DC}} + A\sin(f_\mathrm{d}t)$ and assuming an exponential dependence of J on the barrier voltage, we obtain $J_\mathrm{AC} \propto \exp(A\sin(f_\mathrm{d}t))$. In Fig. S3a, we plot the resulting J_AC modulation profile. This leads to a rectification of the sinusoidal signal, reducing the magnitude of the valleys while amplifying the peaks. Consequently, the exchange modulation amplitude A_J is non-linearly dependant on the amplitude of the drive pulse A. This behavior is further supported by simulations (see Fig. S3b,c) of the resonantly-driven singlet-triplet (ST) qubit's Rabi oscillations, performed using the open-source QuTiP (Quantum Toolbox in Python) software, which qualitatively confirms the observed non-linear dependence.

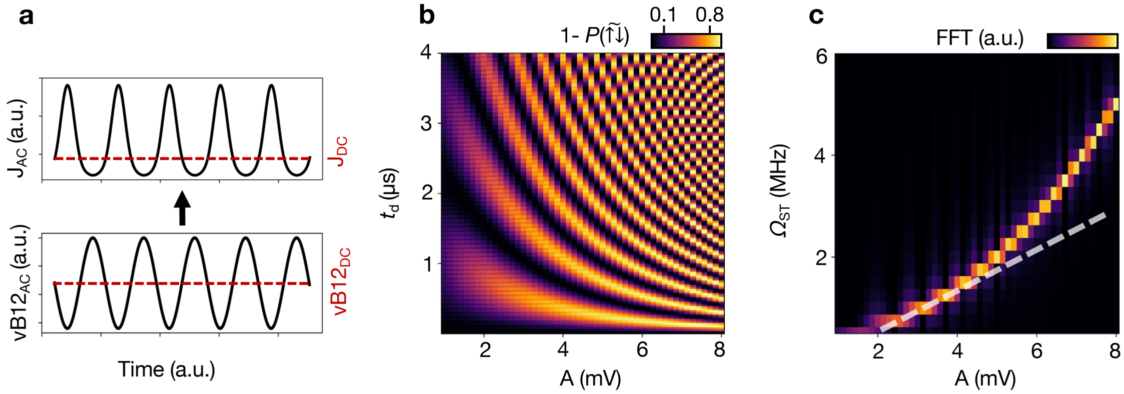

Figure S3. **Resonant ST supplementary plots. a.** Comparison between the shape of the signal applied to the barrier gate (sinus) and the modulation of the exchange interaction. **b.** QuTiP simulation of the dependence of the Rabi oscillation of the ST qubit on the drive amplitude of vB12, assuming an exponential dependence of J on vB12. **c.** The Fourier transform (in arbitrary units, a.u.) reveals that the Rabi frequency depends non-linearly on the drive amplitude of vB12 confirming the experimental observation.

D. DRESSED QUBIT IN THE ROTATING WAVE APPROXIMATION

In this section, we present the calculations relevant for the dressed qubit frame, and derive some important equations of the main text using the rotating wave approximation.

Lab frame. The effective Hamiltonian of the singlet-triplet sector can be written as

$$H'/h = \frac{\Delta E_z}{2h}\sigma'_z + \frac{J_\mathrm{DC}}{2}\sigma'_x, \tag{S1}$$

where $\sigma_z' = |{\downarrow\uparrow}\rangle\langle{\downarrow\uparrow}| - |{\uparrow\downarrow}\rangle\langle{\uparrow\downarrow}|$. We define the lab frame using the eigenstates of this Hamiltonian, i.e., $\{|\widetilde{\downarrow\uparrow}\rangle, |\widetilde{\uparrow\downarrow}\rangle\}$ with eigenvalues $\pm f_{\mathrm{ST}}/2 = \pm\sqrt{(\Delta E_{\mathrm{z}}/h)^2 + J_{\mathrm{DC}}^2}/2$ respectively.

Adding a driven exchange term to Eq. (S1) of the same form with amplitude J'_{AC} we get

$$H/h = \frac{f_{\mathrm{ST}}}{2}\sigma_z + \frac{J'_{\mathrm{AC}}\cos(\omega t)}{2}\left[\frac{\Delta E_{\mathrm{z}}}{hf_{\mathrm{ST}}}\sigma_x + \frac{J_{\mathrm{DC}}}{f_{\mathrm{ST}}}\sigma_z\right], \tag{S2}$$

in the lab frame where $\sigma_z = |\widetilde{\downarrow\uparrow}\rangle\langle\widetilde{\downarrow\uparrow}| - |\widetilde{\uparrow\downarrow}\rangle\langle\widetilde{\uparrow\downarrow}|$. We note that a sinusoidal drive on the barrier gate would induce an oscillating exchange of the form $\mathrm{e}^{A\cos\omega t} \approx I_0(A) + 2I_1(A)\cos(\omega t) + 2I_2(A)\cos(2\omega t) + \ldots$, where $I_n(A)$ is the modified Bessel function of the first kind of order n evaluated at A. Since the upper harmonics in the Fourier series are off-resonant and have a lower amplitude than the first one, we lump the zeroth Fourier component $I_0(A)$ into J_{DC} and consider only the first component $\propto \cos(\omega t)$ for the drive. At a higher order, $J_{\mathrm{DC}} \to J_{\mathrm{DC}}(A)$ leads to a change in the idling *versus* driven qubit frequency that is smaller than the qubit linewidth, and therefore we neglect it. Furthermore, during all RB experiments, the waveforms are assembled without any gaps between the gates, so this effect is avoided altogether. Alternatively, one could in principle "easily" correct for this by tracking the qubit phase shift and applying phase corrections.

Defining the exchange drive along σ_x as $A_J = J'_{\mathrm{AC}}\Delta E_{\mathrm{z}}/hf_{\mathrm{ST}}$ and the second term of the exchange drive along σ_z as $A_{Jz} = J'_{\mathrm{AC}}J_{\mathrm{DC}}/hf_{\mathrm{ST}}$, we arrive at

$$H_{\mathrm{res}}^{\mathrm{ST}}/h = \frac{f_{\mathrm{ST}}}{2}\sigma_z + \frac{J_{\mathrm{AC}}(t)}{2}\sigma_x + \frac{J_{\mathrm{ACz}}(t)}{2}\sigma_z, \tag{S3}$$

where $J_{\mathrm{AC}}(t) = A_J\cos(\omega_d t)$ and $J_{\mathrm{ACz}}(t) = A_{Jz}\cos(\omega_d t)$ with $\omega_d = 2\pi f_d$. We note that for the values of $J_{\mathrm{DC}} \approx 6\,\mathrm{MHz}$ and $\Delta E_{\mathrm{z}}/h \approx 16\,\mathrm{MHz}$ we get $A_J \approx 3A_{Jz}$. Below, we will see that the $J_{\mathrm{ACz}}(t)\sigma_z$ term can be omitted and therefore we arrive at Eq. (S1) of the main text.

Rotating frame. The time-dependent Schrödinger equation with the Hamiltonian of Eq. (S2) can be solved in the RWA, where the solution is given by $|\Psi(t)\rangle = U(t)|\Psi_R(t)\rangle$, where $U(t) = e^{-i\omega_d t\sigma_z/2}$ and $|\Psi_R(t)\rangle$ evolves with the Hamiltonian $H_{\mathrm{RWA}} = U^\dagger(t)HU(t) - \frac{\hbar\omega_d}{2}\sigma_z$.

Let us consider the transformation of the Hamiltonian Eq. (S2) with $U(t)$ term by term:

$$U^\dagger(t)\sigma_z U(t) = \sigma_z, \tag{S4}$$

$$U^\dagger(t)\cos(\omega_d t)\sigma_x U(t) = \frac{1+\cos(2\omega_d t)}{2}\sigma_x - \frac{\sin(2\omega_d t)}{2}\sigma_y \approx \frac{1}{2}\sigma_x, \tag{S5}$$

$$U^\dagger(t)\cos(\omega_d t)\sigma_z U(t) = \cos(\omega_d t)\sigma_z \approx 0, \tag{S6}$$

where we neglected the rapidly oscillating terms ($\cos(2\omega_d t)\sigma_x$, $\sin(2\omega_d t)\sigma_y$ and $\cos(\omega_d t)\sigma_z$) from the rotating-frame Hamiltonian, assuming $|2\pi f_{\mathrm{ST}} - \omega_d| \ll \omega_d$. Finally, we get

$$H_{\mathrm{RWA}}/h = \frac{f_{\mathrm{ST}} - f_d}{2}\sigma_z + \frac{A_J}{4}\sigma_x. \tag{S7}$$

On resonance, i.e., $f_d = f_{\mathrm{ST}}$, the state $|\Psi_R(t)\rangle$ is rotating around the X axis of the rotating frame with the Rabi frequency $\Omega_{\mathrm{ST}} = A_J/2$. This rotation is described by the Hamiltonian

$$H_{\mathrm{RWA}}/h = \frac{\Omega_{\mathrm{ST}}}{2}\tau_z + \frac{f_d - f_{\mathrm{ST}}}{2}\tau_x, \tag{S8}$$

where we define the Pauli matrices of the rotating frame as $\tau_z = \sigma_x = |\tilde{T}_{0R}\rangle\langle\tilde{T}_{0R}| - |\tilde{S}_R\rangle\langle\tilde{S}_R|$, $\tau_x = -\sigma_z$ and $\tau_y = \sigma_y$.

Mollow triplet. In the rotating frame, we can discuss the three resonances of the Mollow triplet. To do this, we add another driving term (probe) to Eq. (S2) with $J'_p\cos((\omega_d + \Delta\omega)t)$ with $\Delta\omega = 2\pi\Delta f$. Moving to the rotating frame the second driving term can be approximated as

$$U^\dagger(t)\cos((\omega_d + \Delta\omega)t)\sigma_x U(t) \approx \frac{\cos(\Delta\omega t)}{2}\sigma_x + \frac{\sin(\Delta\omega t)}{2}\sigma_y, \tag{S9}$$

according to the RWA. When the first tone (pump) is on resonance, i.e., $f_d = f_{\mathrm{ST}}$, the two-tone RWA Hamiltonian then reads as

$$H_{\mathrm{RWA}}/h = \frac{\Omega_{\mathrm{ST}}}{2}\tau_z + \frac{A_J^{\mathrm{probe}}\cos(\Delta\omega t)}{4}\tau_z + \frac{A_J^{\mathrm{probe}}\sin(\Delta\omega t)}{4}\tau_y, \tag{S10}$$

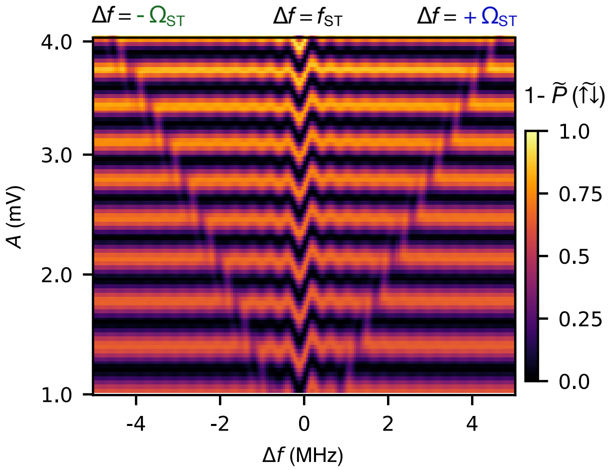

Figure S4. **Mollow triplet.** Simulation of the two-tone driven singlet-triplet qubit using the experimental parameters. The phase of the second tone is shifted by $\pi/4$ to get better agreement with the experimental data around $f_{\mathrm{probe}} \approx f_d$. The reason for this phase shift is not well understood.

where $A_J^{\mathrm{probe}} = J'_{\mathrm{p}} \Delta E_{\mathrm{z}}/hf_{\mathrm{ST}}$ is the amplitude of the second tone. The first driving term would not survive in the RWA, and we can use the fact that the driving terms $\sin(\omega t)\sigma_y$ and $\cos(\omega t)\sigma_x$ are equivalent in the RWA to arrive at

$$H_{\mathrm{RWA}}/h = \frac{\Omega_{\mathrm{ST}}}{2}\tau_z + \frac{A_J^{\mathrm{probe}}\cos(\Delta\omega t)}{4}\tau_x, \tag{S11}$$

which is the same as Eq. (S2) of the main text with $\Delta\nu(t) = A_J^{\mathrm{probe}}\cos(\Delta\omega t)/2$.

We can immediately see that for $\Delta\omega = 0$ the Rabi frequency changes to $\Omega_{\mathrm{ST}} + A_J^{\mathrm{probe}}/2$, which is in good agreement with Fig. 3a. To explain the other two resonances of the Mollow triplet, we can make yet another RWA, exploiting that $A_J^{\mathrm{probe}} \ll A_J$ is satisfied in the experimental range of pump amplitudes. The states of the rotating frame can be recovered from the dressed-frame states as $|\Psi_R(t)\rangle = U_2(t)\,|\Psi_{R,2}(t)\rangle$ using $U_2(t) = e^{-i|\Delta\omega|t\tau_z/2}$. For $\Delta\omega/2\pi \approx \pm\Omega_{\mathrm{ST}}$ the RWA Hamiltonian in the doubly rotating frame becomes

$$H_{\mathrm{RWA},2}/h = \frac{\Omega_{\mathrm{ST}} - |\Delta f|}{2}\tau_z + \mathrm{sgn}(\Delta f)\frac{A_J^{\mathrm{probe}}}{8}\tau_x. \tag{S12}$$

The simulation in Fig. S4 of the full time evolution clearly shows each of these three features at $\Delta f = 0, \pm\Omega_{\mathrm{ST}}$. A non-linear relationship between the barrier drive amplitude and the exchange drive amplitude was assumed.

Frequency modulation. The dressed qubit basis states can also be driven without a second drive tone by modulating the frequency of the drive in Eq. (S8) as

$$f_d(t) = f_{\mathrm{ST}} + \Delta\nu_{\mathrm{FM}}\cos(2\pi f_{\mathrm{FM}}t + \phi_{\mathrm{FM}}), \tag{S13}$$

which provides an equivalent driving mechanism to the two-tone drive. We can also see this from the Fourier spectrum of the frequency-modulated drive which has three peaks around $f \in \{f_{\mathrm{ST}}, f_{\mathrm{ST}} \pm f_{\mathrm{FM}}\}$, while the two-tone drive has two peaks at $f \in \{f_{\mathrm{ST}}, f_{\mathrm{ST}} + \Delta f\}$. In this spectrum the second (smaller) peak provides the driving term for the dressed qubit.

E. DRESSED ST QUBIT

The characteristic Rabi chevron patterns for frequency detunings ($\Delta\nu_{\mathrm{FM}}$) of 1 MHz and 2 MHz are shown in Fig. S5a. When driving with $\Delta\nu_{\mathrm{FM}} = 1$ MHz, we observe that the oscillations begin to fade at higher modulation frequencies (f_{FM}), a phenomenon that may be related to a breakdown of the RWA. However, this effect does not impact the resonant drive at $f_{\mathrm{FM}} = 4.2$ MHz. By introducing an additional phase (ϕ_{FM}) into the frequency-modulated signal, we can rotate the drive axis on the dressed Bloch sphere, similarly to the resonantly-driven ST qubit. Fig. S5d shows this effect, where the axis direction is controlled by ϕ_{FM}. When ϕ_{FM} equals π, $\frac{\pi}{2}$, $\frac{3\pi}{2}$, and similar values, the state and drive axis align, preventing rotations, thereby verifying the effect of the Y^{FM} gate on the dressed Bloch sphere.

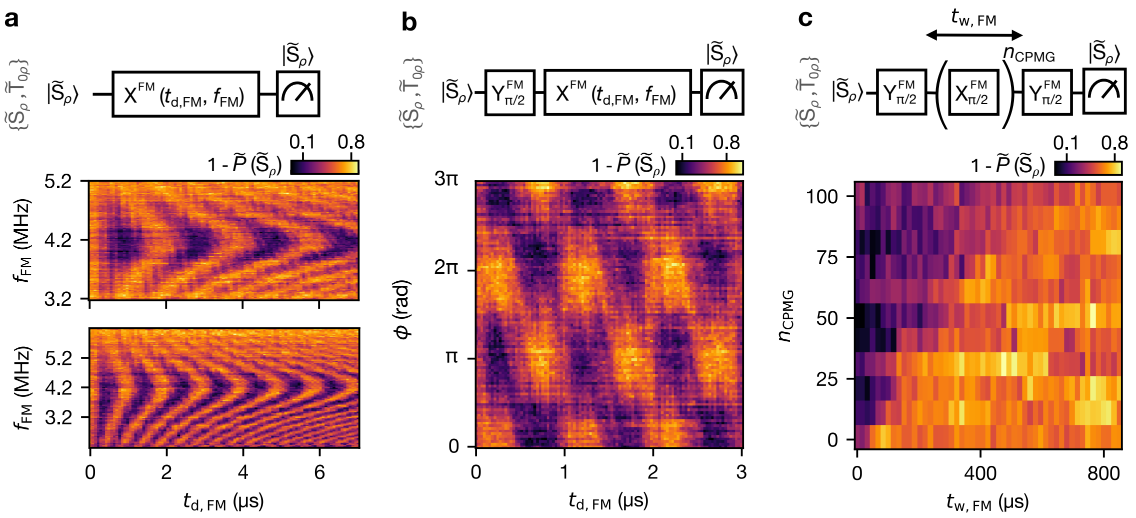

Figure S5. **Dressed singlet-triplet. a.** The Rabi chevron patterns for the FM driven dressed ST qubit for frequency amplitudes ($\Delta\nu_{\mathrm{FM}}$) of 1 MHz and 2 MHz. **b.** By introducing an additional phase (ϕ_{FM}) we demonstrate the rotation of the precession axis, allowing us to achieve both Y and X gates. **c.** CPMG sequence of the dressed ST qubit. Above each plot, the corresponding circuit diagram is illustrated.

We use Y and X pulses to perform a spin echo experiment in Fig. 3 of the main text. By introducing additional X_π pulses, we extend this here to a Carr-Purcell-Meiboom-Gill (CPMG) sequence. Fig. S5c presents the results using up to 100 refocusing pulses, yielding coherence times exceeding 400 μs.

F. DRIVE BANDWIDTH OF THE DRESSED ST QUBIT

We expand on the measurement shown in Fig. 3b(ii) of the main text, showing $\Omega_{\mathrm{ST}}^{\mathrm{FM}}$ as a function of $\Delta\nu_{\mathrm{FM}}$. While in the main text we focused on drive bandwidths lower than $\Omega_{\mathrm{ST}}^{\mathrm{FM}}$ in Fig. S6 we show how the system behaves when driven up to $\Delta\nu_{\mathrm{FM}}/2 = 5\,\mathrm{MHz}$. As can be seen from both the quantitative simulation performed by QuTiP and from the measured data for large values of $\Delta\nu_{\mathrm{FM}}/2$, the RWA seems to start breaking as the FFT amplitude fades away and additional frequency components appear. Further study of the dynamics in this regime can be found in Ref. [35] but fall outside of the scope of this study, where we operate the dressed ST qubit at a maximum $\Delta\nu_{\mathrm{FM}} = 1\,\mathrm{MHz}$.

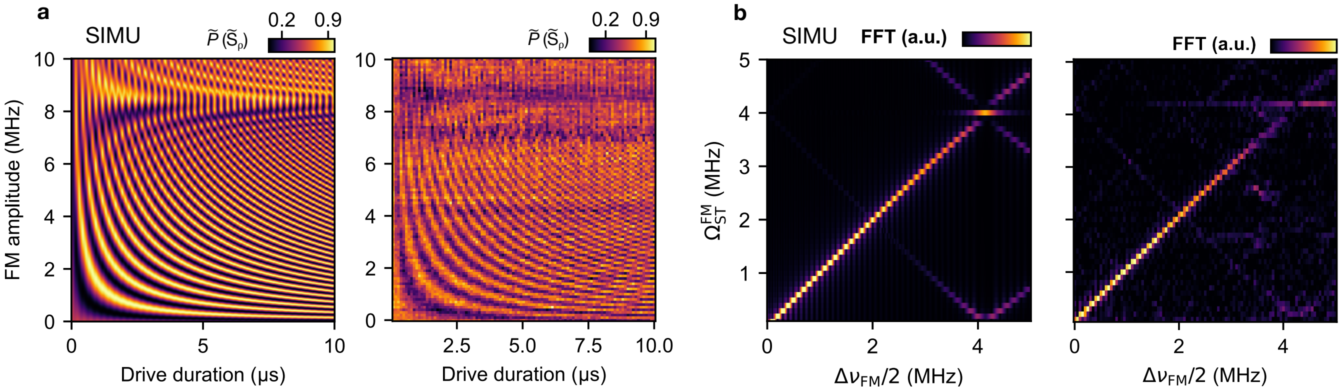

Figure S6. **Dressed ST qubit drive bandwith. a.** Dressed ST qubit driving for different values of $\Delta\nu_{\mathrm{FM}}$: QuTiP simulations (left) and experimental measurements (right). **b.** The FFT (in arbitrary units, a.u.) along the time axis [QuTiP simulations (left) and experimental measurements (right)] revealing the dependence of the dressed Rabi frequency $\Omega_{\mathrm{ST}}^{\mathrm{FM}}$ on $\Delta\nu_{\mathrm{FM}}$.

G. VIRTUAL MATRIX

The virtual gate matrix is shown in Tab. S1.

Table S1. **Virtual gate matrix.**

	vP1	vP2	vP3	vP4	vP5	vP6	vB12	vB23	vB34	vB45	vB56	vBO1	vB6O	vBM	vS1P	vS1B1	vS1B2
P1	1.0	0	0	0	0	0	0	0	0	0	0	0	0	0	0	0	0
P2	0	1.0	0	0	0	0	0	0	0	0	0	0	0	0	0	0	0
P3	0	0	1.0	0	0	0	0	0	0	0	0	0	0	0	0	0	0
P4	0	0	0	1.0	0	0	0	0	0	0	0	0	0	0	0	0	0
P5	0	-0.015	-0.049	-0.14	1.0	-0.18	0	-0.03	-0.15	-0.575	-0.56	0	-0.05	-0.45	0	0	0
P6	0	0	-0.02	-0.075	-0.2	1.0	0	-0.013	-0.03	-0.075	-0.65	0	-0.14	-0.2	0	0	0
B12	0	0	0	0	0	0	1.0	0	0	0	0	0	0	0	0	0	0
B23	0	0	0	0	0	0	0	1.0	0	0	0	0	0	0	0	0	0
B34	0	0	0	0	0	0	0	0	1.0	0	0	0	0	0	0	0	0
B45	0	0	0	0	0	0	0	0	0	1.0	0	0	0	0	0	0	0
B56	0	0	0	0	0	0	0	0	0	0	1.0	0	0	0	0	0	0
BO1	0	0	0	0	0	0	0	0	0	0	0	1.0	0	0	0	0	0
B6O	0	0	0	0	0	0	0	0	0	0	0	0	1.0	0	0	0	0
BM	0	0	0	0	0	0	0	0	0	0	0	0	0	1.0	0	0	0
S1P	-0.005	-0.004	-0.01	-0.027	-0.044	-0.025	-0.013	-0.029	-0.028	-0.02	0	0	-0.003	-0.4	1.0	0	0
S1B1	0	0	0	0	0	0	0	0	0	0	0	0	0	0	0	1.0	0
S1B2	0	0	0	0	0	0	0	0	0	0	0	0	0	0	0	0	1.0
